# HPV-positive murine oral squamous cell carcinoma: development and characterization of a new mouse tumor model for immunological studies

**DOI:** 10.1186/s12967-023-04221-4

**Published:** 2023-06-10

**Authors:** Ziva Modic, Maja Cemazar, Bostjan Markelc, Andrej Cör, Gregor Sersa, Simona Kranjc Brezar, Tanja Jesenko

**Affiliations:** 1 Department of Experimental Oncology, Institute of Oncology Ljubljana, Zaloska cesta 2, Ljubljana, Slovenia; 2 Faculty of Medicine, University of Ljubljana, Vrazov trg 2, Ljubljana, Slovenia; 3 Faculty of Health Sciences, University of Primorska, Polje 42, Izola, Slovenia; 4 Faculty of Health Sciences, University of Ljubljana, Zdravstvena pot 5, Ljubljana, Slovenia; 5 Department of Research, Valdoltra Orthopedic Hospital, Jadranska cesta 31, Ankaran, Slovenia; 6 Faculty of Education, University of Primorska, Cankarjeva pot 5, Koper, Slovenia

**Keywords:** Syngeneic mouse model, Immunocompetent mouse model, Oral squamous cell carcinoma, Human papillomavirus

## Abstract

**Background:**

Infection with high-risk human papillomavirus (HPV) strains is one of the risk factors for the development of oral squamous cell carcinoma (OSCC). Some patients with HPV-positive OSCC have a better prognosis and respond better to various treatment modalities, including radiotherapy or immunotherapy. However, since HPV can only infect human cells, there are only a few immunocompetent mouse models available that enable immunological studies. Therefore, the aim of our study was to develop a transplantable immunocompetent mouse model of HPV-positive OSCC and characterize it in vitro and in vivo.

**Methods:**

Two monoclonal HPV-positive OSCC mouse cell lines were established by inducing the expression of HPV-16 oncogenes E6 and E7 in the MOC1 OSCC cell line using retroviral transduction. After confirming stable expression of HPV-16 E6 and E7 with quantitative real-time PCR and immunofluorescence staining, the cell lines were further characterized in vitro using proliferation assay, wound healing assay, clonogenic assay and RNA sequencing. In addition, tumor models were characterized in vivo in C57Bl/6NCrl mice in terms of their histological properties, tumor growth kinetics, and radiosensitivity. Furthermore, immunofluorescence staining of blood vessels, hypoxic areas, proliferating cells and immune cells was performed to characterize the tumor microenvironment of all three tumor models.

**Results:**

Characterization of the resulting MOC1-HPV cell lines and tumor models confirmed stable expression of HPV-16 oncogenes and differences in cell morphology, in vitro migration capacity, and tumor microenvironment characteristics. Although the cell lines did not differ in their intrinsic radiosensitivity, one of the HPV-positive tumor models, MOC1-HPV K1, showed a significantly longer growth delay after irradiation with a single dose of 15 Gy compared to parental MOC1 tumors. Consistent with this, MOC1-HPV K1 tumors had a lower percentage of hypoxic tumor area and a higher percentage of proliferating cells. Characteristics of the newly developed HPV-positive OSCC tumor models correlate with the transcriptomic profile of MOC1-HPV cell lines.

**Conclusions:**

In conclusion, we developed and characterized a novel immunocompetent mouse model of HPV-positive OSCC that exhibits increased radiosensitivity and enables studies of immune-based treatment approaches in HPV-positive OSCC.

**Supplementary Information:**

The online version contains supplementary material available at 10.1186/s12967-023-04221-4.

## Background

Oral squamous cell carcinoma (OSCC) includes malignant neoplasms of the lip, oral cavity and oropharynx. In 2020, approximately 480,000 cases of OSCC were diagnosed worldwide, representing close to 2.5% of all diagnosed malignancies [1]. Standard approaches for the treatment of locally confined OSCC include resection, radiotherapy, and chemoradiotherapy. For recurrent or metastatic disease, systemic approaches such as chemotherapy and immunotherapy with cetuximab or immune checkpoint inhibitors are used [2]. The main risk factors for the development of OSCC are alcohol and tobacco consumption [3, 4]. Additionally, several epidemiological studies have shown that persistent infection with high-risk human papillomavirus (HPV) strains correlates with an increased risk for the development of oropharyngeal OSCC. High-risk HPV DNA was also detected with increased frequency in malignant lesions of the oral epithelium; however, the role of HPV in the development of non-oropharyngeal OSCC is not yet clear [5–10]. Moreover, clinical data indicate that patients with HPV-positive OSCC can have a better prognosis [9, 11, 12] with a better response to treatment with chemo-radiotherapy [13], radiotherapy alone [14, 15] or immunotherapy with immune checkpoint inhibitors [16].

HPVs are separated into low-risk and high-risk strains based on their oncogenic potential. Out of more than 200 identified HPV strains, 12 have been recognized as high risk [17]. Among those, the most important for OSCC development are HPV-16 and HPV-18, which contribute to 85% of all HPV-positive head and neck cancers [18]. The main factors associated with HPV carcinogenesis are viral oncogenes E6 and E7, which induce ubiquitination and proteasomal degradation of two key tumor-suppressing proteins, p53 and retinoblastoma protein (pRb), respectively [19, 20], leading to uncontrolled DNA replication, limited DNA damage response, and consequently genome instability [21–23]. Importantly, high-risk HPV DNA can also integrate into the host cell genome through integration breakpoints that commonly occur in the viral genes E1 and E2, which are responsible for the regulation of viral oncogene expression, resulting in elevated expression levels and stabilization of E6 and E7 mRNA [24–26].

Although a significant proportion of OSCCs are associated with HPV infection, there is a lack of preclinical models suitable for studies of these mechanisms and immunological studies. In recent years, great progress has been made in the development of innovative in vitro approaches for 3D modelling of HPV-positive and HPV-negative OSCC (reviewed in [27, 28]) which have several advantages over conventional adherent 2D cell cultures, such as better replication of the tumor tissue architecture and physical properties of tumor microenvironment. Culturing of human OSCC cell lines or primary cells on collagen-based 3D scaffolds enables the crosstalk between cancer cells and extracellular matrix, which mimics the tumor microenvironment [29]. Tumor tissue has also been cultured in the form of histocultures, in which primary tumor tissue is excised and cultured ex vivo. Although this method enables co-culturing of cancer cells in their original microenvironment with immune, stromal and vascular cells, the short lifespan of such cultures is a limiting factor. Engelmann et al. [30] used supportive scaffolds of healthy human-derived fibroblasts and viscose fibers to prolong the proliferation of the tumor cells in histocultures to up to 21 days. In addition, microfluidic chips have been used to co-culture OSCC cells with immune cells to study the effect of immunotherapies [31, 32]. Nevertheless, immunocompetent in vivo mouse models are still commonly used and needed in research of novel immune-based treatment approaches. Since HPV can only infect human cells, there is a lack of immunocompetent mouse models of HPV-positive OSCC. The majority of the in vivo models used in preclinical research are xenograft mouse models using cells originating from patient-derived HPV-positive tumors transplanted into immunodeficient athymic nude mice, which are not suitable for immunologic studies [33–37].

Therefore, the goal of our study was to develop and characterize a novel transplantable immunocompetent mouse model of HPV-positive OSCC that would resemble the clinical features of HPV-positive OSCC. Such a model would enable studies of the immunological components of currently employed treatments and evaluation of novel treatment approaches that are based on mechanisms of adaptive immunity in both HPV-positive and HPV-negative models of OSCC. The proposed model was developed through stable integration of the E6 and E7 oncogenes into the genome of a mouse OSCC cell line using retroviral transduction and further characterized in vitro and in vivo.

## Methods

### Establishment of HPV-positive OSCC cell line

An HPV-positive OSCC cell line was established by transduction of the murine OSCC cell line MOC1 with LXSN16E6E7 retroviral particles encoding the HPV-16 E6 and E7 genes along with a gene for neomycin resistance. After antibiotic selection (600 µg/mL G418 disulfate solution (AppliChem, Darmstadt, Germany)) for transduced cells, monoclonal cell lines were established. Monoclonal cell lines used in this study were named MOC1-HPV K1 and MOC1-HPV K3 and were cultured under standard conditions (37 °C, 5% CO_2_ humidified atmosphere) in MOC1 medium (Iscove's Modified Dulbecco's Medium (IMDM)/Ham’s F12 Nutrient Mixture (Gibco, Thermo Fisher Scientific, Waltham, MA, USA), in a ratio of 2:1, supplemented with 5% (v/v) fetal bovine serum (FBS, Gibco), 100 U/mL penicillin, 100 µg/mL streptomycin (100 × penicillin‒streptomycin, Sigma Aldrich, Darmstadt, Germany), 5 µg/mL insulin (Sigma Aldrich), 40 ng/mL hydrocortisone (Sigma Aldrich) and 5 ng/mL epidermal growth factor (EGF, Gibco)) with added 200 µg/mL G418 disulfate. To confirm the stable expression of HPV-16 E6 and E7, RNA was extracted using a peqGOLD Total RNA Kit (VWR, West Chester, PA, USA) following the manufacturer’s instructions, and qRT‒PCR for E6, E7, and housekeeping genes (GAPDH and β-actin) was performed. Immunofluorescence staining was used to confirm the expression of E6 and E7 at the protein level.

### In vitro characterization of the newly established cell lines

#### Cell proliferation assay

To determine the proliferation kinetics of the cell lines, PrestoBlue™ Cell Viability Reagent (Invitrogen, Thermo Fisher Scientific) was performed on four consecutive days according to the manufacturers’ instructions.

#### Wound healing assay

Cell migration capacity was assessed by wound-healing assay using 24-well plates with two-well silicone inserts (Ibidi, Gräfelfing, Germany).

#### Radiosensitivity evaluation in vitro

To determine the radiosensitivity of the cell lines, cells were irradiated, and a clonogenic assay was performed.

#### Transcriptome analysis

For transcriptome analysis RNA-sequencing was performed on four biological replicates of each cell line. In addition, to compare the transcriptomic profile of murine MOC1-HPV cell lines with patient data, transcriptome analysis of publicly available data from four HPV-positive (UM-SCC-104, UM-SCC-47, and UPC-SCC-090) and one HPV-negative cell line (FaDu), as well as HPV-positive and HPV-negative patient tumor samples (NCBI Gene Expression Omnibus (GEO) series accession number GSE211322) [38] was carried out. NCBI-generated counts data of GSE211322 samples was downloaded and analyzed using free online tool iDEP.951 [39].

### In vivo characterization of MOC1-HPV tumor models

#### Animals and tumor induction

All experiments were approved by the Ministry of Agriculture, Forestry and Food of the Republic of Slovenia (permission no. U34401-35/2020/8). Up to six mice were housed per cage in specific-pathogen-free conditions in a carousel mouse IVC rack system (Animal Care Systems Inc., Revere Parkway, USA) with a 12 h light–dark cycle, food and water ad libitum, and cage enrichment (VWR). To induce tumor growth 1 × 10^6^ MOC1, MOC1-HPV K1, or MOC1-HPV K3 cells were injected subcutaneously into the flank of 8- to 12-week-old female C57Bl/6NCrl mice (Charles River, Lecco, Italy).

#### Determination of HPV-16 E6 and E7 in vivo

Expression of HPV-16 E6 and E7 was confirmed by quantitative real-time PCR (qRT‒PCR) in tumors at volumes of 50–60 mm^3^.

#### Tumor histology

Tumors were collected at 50–60 mm^3^ or 100 mm^3^, fixed in 10% neutral buffered formalin and embedded in paraffin. Paraffin sections (2-µm-thick) were stained with hematoxylin and eosin (HE), and evaluated by an experienced pathologist.

#### Tumor microenvironment assessment

Characteristics of the tumor microenvironment were assessed on tumors at 50–60 mm^3^. Three hours before the animals were sacrificed, EdU (Abcam, Cambridge, United Kingdom) and EF5 (EF5 Hypoxia Detection Kit, Cyanine 3; EMD Millipore, CA, USA) were injected intraperitoneally to mark the proliferating cells and hypoxic areas, respectively. Collected tumors were fixed in 4% PFA and dehydrated in 30% sucrose before embedding in Tissue-Tek O.C.T. compound (Sakura Finetek, VWR). Fourteen µm thick frozen tissue sections were then immunofluorescently labeled for EdU, EF5, CD31 (R&D Systems, MN, USA), CD4 (Abcam), CD8 (Abcam) and F4/80 (Thermo Fisher Scientific). Nuclei were counterstained with Hoechst 33343 pentahydrate (Thermo Fisher Scientific).

#### Radiosensitivity evaluation in vivo

To evaluate the radiosensitivity of tumors in vivo, mice were randomly assigned to the control or irradiated group when the tumors reached 50–60 mm^3^ using a Graphpad QuickCalcs random number generator. The irradiated group received a single irradiation dose of 15 Gy. Tumor growth was evaluated by determining the tumor volume three times weekly using the formula for ellipsoid (V = a × b × c × π/6, where a, b, and c are perpendicular tumor diameters). Animal weight was monitored three times a week as a sign of wellbeing. Growth delay was calculated by determining the time irradiated tumors needed to grow from 50–60 mm^3^ to 100 mm^3^ (time to 100 mm^3^) and subtracting the time to 100 mm^3^ of control tumors.

### Statistical analysis

Statistical analysis and graph plotting were performed using GraphPad Prism 9 (La Jolla, CA, USA). Datasets were tested for normal distribution using the D’Agostino-Pearson normality test. Statistical significance was evaluated using ordinary one-way ANOVA with Tukey’s multiple comparisons test for normally distributed data and nonparametric one-way ANOVA (Kruskal‒Wallis test) for data without a normal distribution. A p < 0.05 was considered statistically significant. Throughout the manuscript, the following symbols indicate statistical significance: *p < 0.05, **p < 0.01, ***p < 0.001, and ****p < 0.0001. The sample size (n) for each experiment is presented in the figure legends and represents the number of biological replicates unless otherwise stated. All experiments were repeated at least two times.

A detailed description of the methods is included in the Additional file 2: Supplementary methods.

## Results

### Establishment of HPV-16 E6- and E7-expressing cell lines MOC1-HPV

**Fig. 1:**
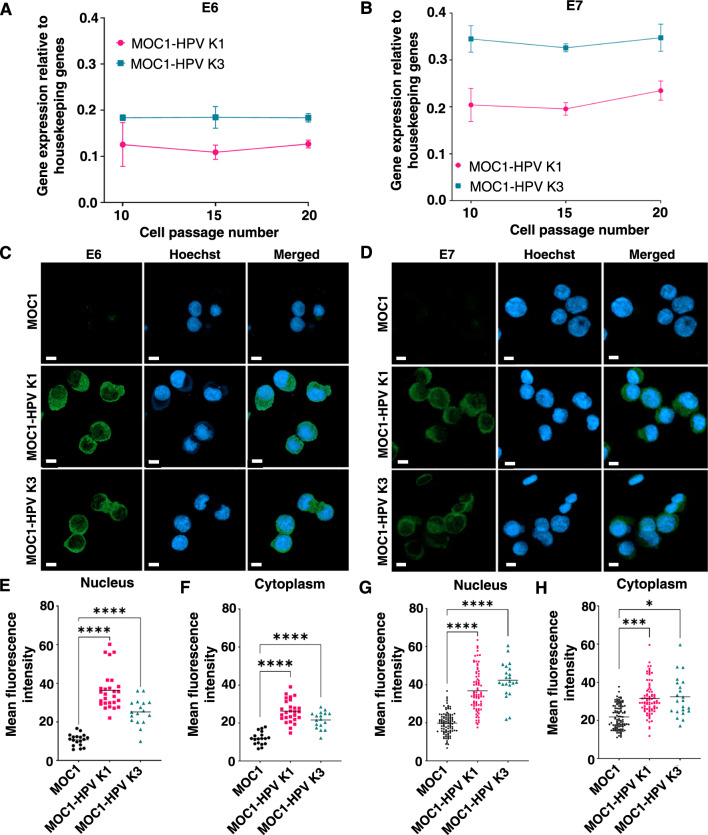
MOC1-HPV monoclonal cell lines express HPV-16 E6 and E7 at the RNA and protein levels. **A**,** B** Expression of HPV-16 E6 (**A**) and E7 (**B**) at the mRNA level remains stable with cell passaging as determined by qRT‒PCR. Housekeeping genes: GAPDH and β-actin. The data are presented as the mean ± SD. n = 2. **C**, **D** Representative images of cells immunofluorescently stained for HPV-16 E6 (**C**) or HPV-16 E7 (**D**). Scale bar: 10 µm. **E**–**H** Mean fluorescence intensity of E6 (**E**, **F**) and E7 (**G**, **H**) in the nucleus and cytoplasm. Dot plots present all data with the black line representing the mean. Up to 100 cells of each cell line were analyzed. *p < 0.05, ***p < 0.001, ****p < 0.0001, one-way ANOVA

To establish an HPV-positive OSCC cell line, we inserted the HPV-16 E6 and E7 oncogenes into the genome of MOC1 cells by retroviral transduction, as expression of the E6 and E7 oncogenes was shown to be sufficient for cell transformation [40, 41]. Using qRT‒PCR, we confirmed that insertion of HPV-16 E6 and E7 oncogenes into the cell genome was stable in both monoclonal cell lines (MOC1-HPV K1 and MOC1-HPV K3), resulting in stable expression of E6 and E7 mRNA across at least 20 cell passages (Fig. 1A, B). Furthermore, stability of the overall gene expression in MOC1-HPV cell lines is also evident from RNA sequencing results (Additional file 1: Fig. S1). Expression of E6 and E7 proteins was also confirmed by immunofluorescence staining (Fig. 1C, D). E6 (Fig. 1E, F) and E7 (Fig. 1G, H) were detected in both the nuclei and cytoplasm of the cells, with significantly higher mean fluorescence intensity in both MOC1-HPV cell lines than in the parental MOC1 cell line.

### Morphological examination, cell growth and migration capacity in vitro

**Fig. 2:**
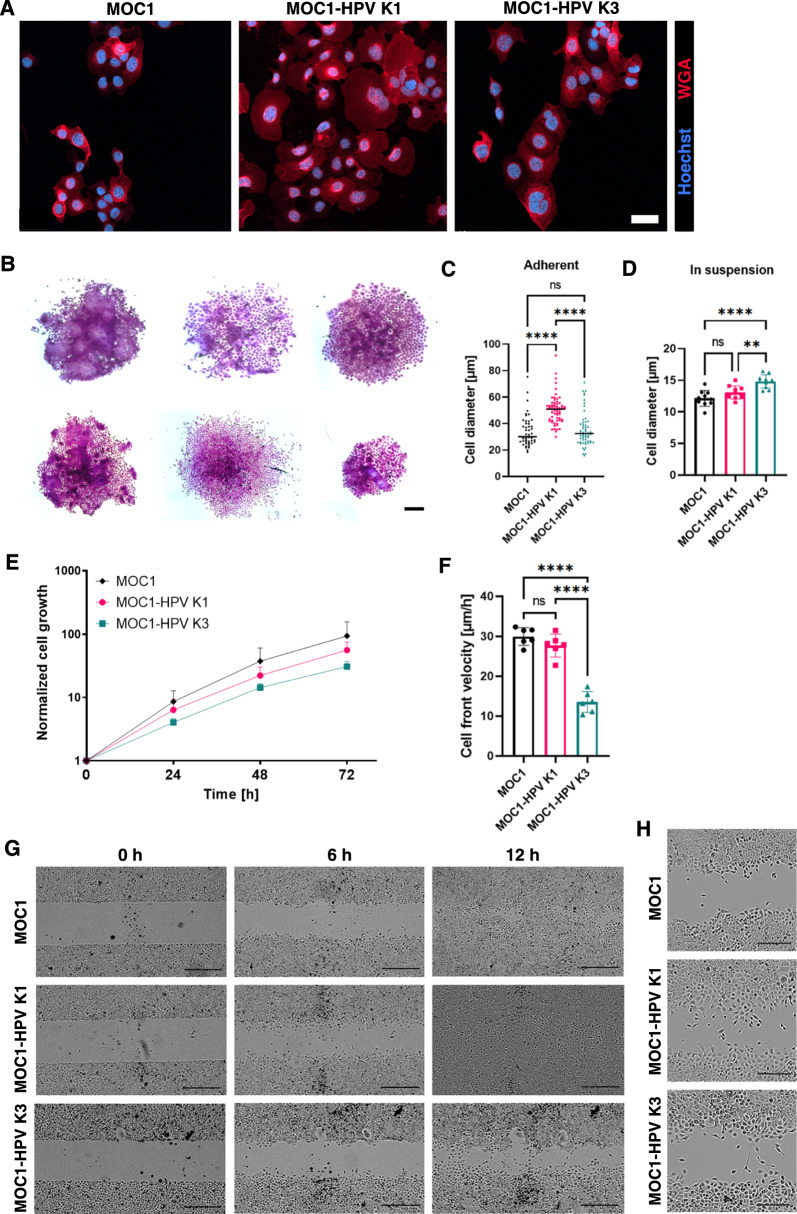
In vitro characterization of MOC1 and MOC1-HPV cell lines. **A** Representative immunofluorescent images of MOC1 and MOC1-HPV cells after staining of the plasma membrane with WGA (red) and nuclei with Hoechst 33342 (blue). Scale bar: 40 µm. **B** Two representative cell colonies of MOC1, MOC1-HPV K1, and MOC1-HPV K3 cells six days after seeding. Scale bar: 200 µm. **C** Average cell diameter of adherent cells. Dot plots present all data with the black line representing the mean. Between 50 and 65 cells of each cell line were analyzed. ****p < 0.0001, ns: not significant, nonparametric one-way ANOVA (Kruskal‒Wallis test). **D** Cell diameter of trypsinized cells in suspension. In total, up to 11,000 cells of each cell line from 10 individual experiments were analyzed. The bar plot presents the mean of each experiment ± SD. **p < 0.01, ****p < 0.0001, *ns* not significant, ordinary one-way ANOVA. **E** Proliferation of MOC1 and MOC1-HPV cell lines. Presented are the mean + SD. n = 3. **F** Cell front velocity of MOC1 and MOC1-HPV cell lines in the wound-healing assay. Presented are the mean ± SD. ****p < 0.0001, ordinary one-way ANOVA. n = 6. **G**, **H** Representative bright-field images of cell migration in the wound-healing assay. Scale bar: 500 µm (**G**) or 250 µm (**H**)

For in vitro characterization of the cell lines, we first focused on cell morphology, cell growth kinetics, and cell migration ability. Cell morphology was examined by confocal and bright-field microscopy. MOC1 and MOC1-HPV K3 cells grew in close contact, whereas MOC1-HPV K1 cells showed less pronounced cell-to-cell contacts (Fig. 2A), which was also reflected in looser MOC1-HPV K1-cell colonies in the clonogenic assay (Fig. 2B). In addition, MOC1-HPV K1 cells appeared larger than MOC1 and MOC1-HPV K3 cells (Fig. 2A) with significantly larger cell diameters when attached (Fig. 2C) but not when in suspension after trypsinization (Fig. 2D), also suggesting differences in cell adhesion properties.

Although MOC1-HPV K3 cells proliferated slower in vitro, the differences in cell growth between the cell lines were not statistically significant (Fig. 2E). To assess the migration potential of the cell lines in vitro, a wound-healing assay was performed (Fig. 2F–H). The cell front velocity was significantly lower in the MOC1-HPV K3 cells (Fig. 2F). While the MOC1 and MOC1-HPV K1 cell lines closed the 500 µm wide cell-free gap approximately 12 h after removal of the inserts, the MOC1-HPV K3 cells required more than 18 h (Fig. 2G). Cells of all three cell lines showed signs of collective migration, with most cells maintaining intercellular connections during migration and wound closure, although looser cell-to-cell contacts were still observed in MOC1-HPV K1 (Fig. 2H). We have also confirmed that the observed phenotype in MOC1-HPV cells with regards to the differences in the migration kinetics is independent of cell passaging, by repeating the wound-healing assay after prolonged culturing of MOC1-HPV cells to reach passages between 17 and 20 (Additional file 1: Fig. S2).

### Transcriptome analysis

**Fig. 3:**
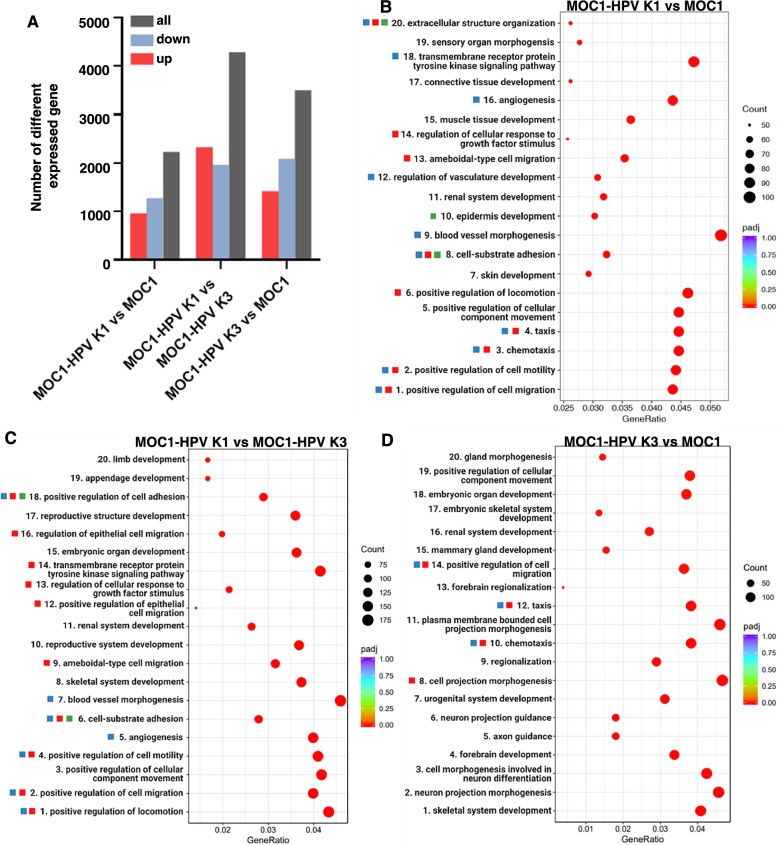
Transcriptome analysis of MOC1 and MOC1-HPV cell lines.** A** Number of differentially expressed genes in MOC1 and MOC1-HPV cell lines. Genes with an adjusted p value < 0.05 and fold change > 1 were considered differentially expressed. **B**–**D** The twenty most significantly enriched GO biological process terms with an adjusted p value < 0.05 comparing MOC1-HPV K1 to MOC1 (**B**), MOC1-HPV K1 to MOC1-HPV K3 (**C**), and MOC1-HPV K3 to MOC1 (**D**) are shown. The position of the dots relates to the GeneRatio, which is the fraction of all differentially expressed genes that are found in the respective GO term. The size and color of the dots represent the number of differentially expressed genes in the pathway and adjusted p value, respectively. Color-coding of the squares next to the biological terms refers to the observed cell phenotype—red: cell migration, blue: angiogenesis, green: cell‒cell contacts

**Fig. 4:**
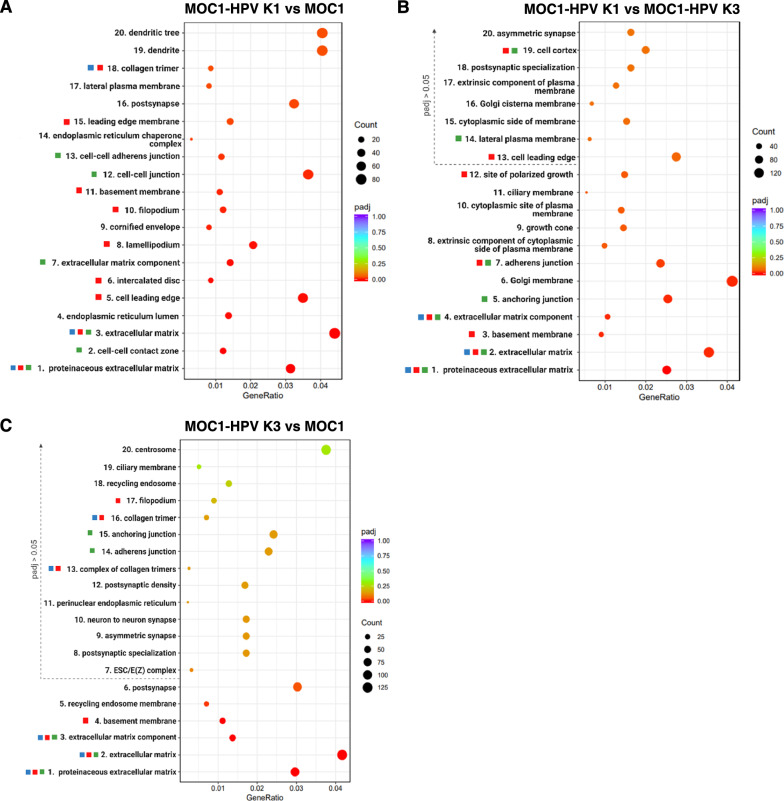
Enriched GO cellular compartment terms in MOC1 and MOC1-HPV cell lines.** A**–**C** The twenty most significantly enriched GO cellular compartment terms comparing MOC1-HPV K1 to MOC1 (**A**), MOC1-HPV K1 to MOC1-HPV K3 (**B**), and MOC1-HPV K1 to MOC1 (**C**) are shown. The position of the dots relates to the GeneRatio, which is the fraction of differentially expressed genes that are found in the respective GO term. The size and color of the dots represent the number of differentially expressed genes in the pathway and adjusted p value, respectively. Color-coding of the squares next to the biological terms refers to the observed cell phenotype—red: cell migration, blue: angiogenesis, green: cell‒cell contacts

Next, we used RNA sequencing to analyze the changes in gene expression in vitro after integration and expression of HPV-16 E6 and E7. The three cell lines showed different gene expression patterns with between 2000 and 4000 differentially expressed genes (Fig. 3A). With Gene Ontology (GO) analysis of the differentially expressed genes, we found that in the MOC1-HPV K1 cell line compared to the MOC1 or MOC1-HPV K3 cell line, the majority of the 20 most enriched GO terms within the “Biological Processes” compartment were related to angiogenesis, taxis, cell migration and motility, cell adhesion, and extracellular matrix organization (Fig. 3B, C), which correlates well with the observed differences in cell morphology and migration. When comparing the MOC1-HPV K3 cell line to the parental MOC1 cell line, the 20 most enriched GO biological process terms were mainly related to developmental processes and morphogenesis but also to cell taxis and migration (Fig. 3D). In terms of GO cellular component terms, the 20 most enriched in the MOC1-HPV K1 cell line both when compared to MOC1 (Fig. 4A) or MOC1-HPV K3 cell line (Fig. 4B) included extracellular matrix and cell junctions, which is in line with morphologic differences observed in vitro. When comparing MOC1-HPV K3 cells to the parental MOC1 cell line, only six significantly enriched GO cellular component terms were identified, which were related to the extracellular matrix, recycling endosome membrane, and synapse (Fig. 4C).

To evaluate the transcriptional resemblance of MOC1-HPV cell lines to transcriptional characteristics of human OSCC tumors, we analyzed publicly available transcriptome data of human patient-derived cell lines of HPV-positive (UM-SCC-104, UM-SCC-47, UPCI-SCC-090) and HPV-negative (FaDu) OSCC and of patient tumor samples (Additional file 1: Tables S1–S4). The most enriched GO pathways when comparing human HPV-positive and HPV-negative cell lines included pathways related to extracellular matrix, angiogenesis, and cell migration, which largely overlapped with the results of the comparison of MOC1-HPV and MOC1 cell lines. On the other hand, the enriched pathways in HPV-positive patient tumors did not overlap with those enriched in MOC1-HPV cell lines, and also to a lesser extent with enriched pathways in human HPV-positive OSCC cell lines.

### MOC1 and MOC1-HPV cell lines induce tumors in C57Bl/6 mice

**Fig. 5:**
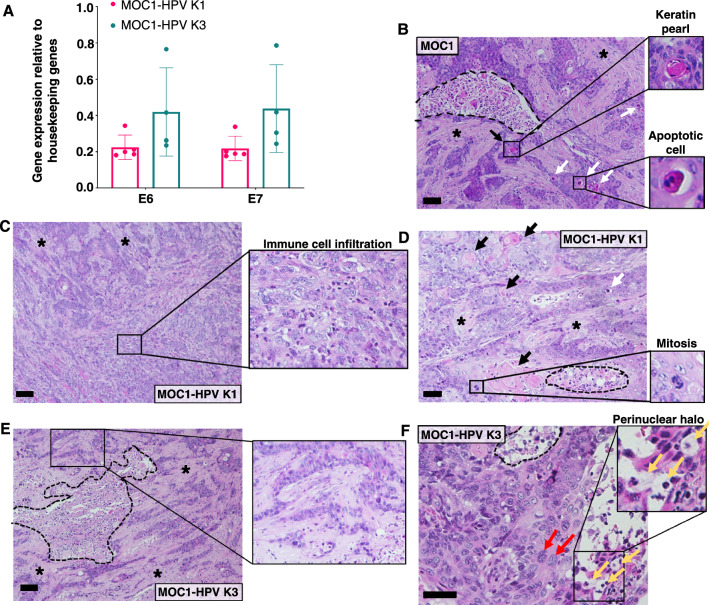
MOC1-HPV tumors express HPV oncogenes E6 and E7 and show histological features of squamous cell carcinomas. **A** Expression of the HPV-16 E6 and E7 oncogenes in tumor tissue was determined at a tumor volume of 50 mm^3^ using qRT‒PCR. Housekeeping genes: GAPDH and β-actin. Presented is the mean ± SD. n = 4–5. **B**–**F** Representative images of hematoxylin and eosin-stained paraffin sections of MOC1 (**B**), MOC1-HPV K1 (**C**, **D**), and MOC1-HPV K3 (**E**, **F**) tumor cores. Tumors were isolated when they reached volumes of 50–100 mm^3^. Dotted lines denote necrotic areas, asterisks denote desmoplasia, black denote arrows keratinization, white arrows denote apoptotic cells, red arrows denote cells with large nuclei, and yellow arrows denote cells with a perinuclear halo. Images **B**–**E** were acquired using a 10 × objective, and image **F** was acquired with a 20 × objective. Scale bar: 100 µm. n = 4

Next, we aimed to characterize the HPV-positive OSCC models in vivo. Subcutaneous injection of 1 × 10^6^ MOC1 or MOC1-HPV cells into the flank of C57Bl/6 mice induced subcutaneous tumors. Palpable tumors were obtained approximately one week after tumor cell injection in approximately 88% of the mice. MOC1 tumors reached a volume of 50–60 mm^3^ in 26.3 ± 4.0 days (min 15, max 46 days), MOC1-HPV K1 tumors in 21.4 ± 7.2 days (min 8, max 48 days), and MOC1-HPV K3 tumors in 15.7 ± 1.8 days (min 10, max 34 days) with comparable tumor growth kinetics between the experiments. Using qRT‒PCR, we confirmed the expression of HPV-16 E6 and E7 mRNA in MOC1-HPV K1 and MOC1-HPV K3 tumors at 50–60 mm^3^ (Fig. 5A). Histological analysis of paraffin sections of MOC1 tumors stained with hematoxylin and eosin showed typical features of differentiated squamous cell carcinoma, such as keratinization and desmoplasia. Parts of the tumor core showed necrotic areas (Fig. 5B). MOC1-HPV K1 tumors exhibited a high degree of immune cell infiltration both at the tumor margin and in the tumor core (Fig. 5C), with few necrotic areas, few apoptotic cells, and some mitotic cells (Fig. 5D). On the other hand, MOC1-HPV K3 tumors showed larger necrotic areas and less immune cell infiltration (Fig. 5E). In addition, several cells with large nuclei and multinuclear cells were found in MOC1-HPV K3 tumors (Fig. 5F). In MOC1-HPV K1 and MOC1-HPV K3 tumors, several cells had a perinuclear halo (Fig. 5F).

### Tumor microenvironment of MOC1 and MOC1-HPV tumors

**Fig. 6:**
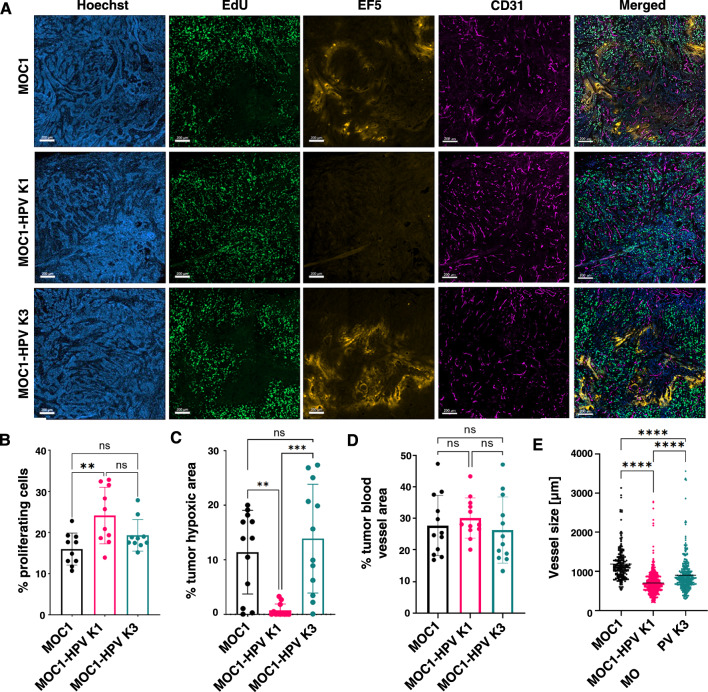
Tumor microenvironment of MOC1 and MOC1-HPV tumors. **A** Representative images of immunofluorescently stained frozen sections of the tumor core. Proliferating cells are marked with EdU (Green), hypoxic regions with EF5 (Orange), blood vessels with CD31 (Magenta), and cell nuclei with Hoechst 33342 (Blue). Scale bar: 200 µm. **B**–**E** The fluorescent signal was quantified and expressed as the percentage of proliferating cells (**B**), percentage of hypoxic area (**C**), percentage of tumor blood vessel area (**D**), and average vessel size (**E**). Bar plots present the mean ± SD. The black line in the dot plot presents the mean. n = 4. *ns* not significant, **p < 0.01, ***p < 0.001, ****p < 0.0001, ordinary one-way ANOVA (**B**–**D**) and nonparametric one-way ANOVA (Kruskal‒Wallis test) (**E**)

Next, we were interested in the tumor microenvironment of the three tumor models, particularly in the extent of hypoxia and proliferating cells. To this end, we immunofluorescently labeled proliferating cells with EdU, hypoxic areas with EF5, and blood vessels with CD31 (Fig. 6A). By quantifying the fluorescence signal, we showed that MOC1-HPV K1 tumors had significantly more proliferating cells than MOC1 and MOC1-HPV K3 tumors (Fig. 6B). Moreover, MOC1-HPV K1 tumors were significantly less hypoxic than MOC1 and MOC1-HPV K3 tumors (Fig. 6C), even though the percentage of blood vessel area was comparable in all three tumor models (Fig. 6D). However, when examining the area of each vessel, the MOC1-HPV K1 tumors had significantly smaller vessels than the MOC1 and MOC1-HPV K3 tumors (Fig. 6E).

**Fig. 7:**
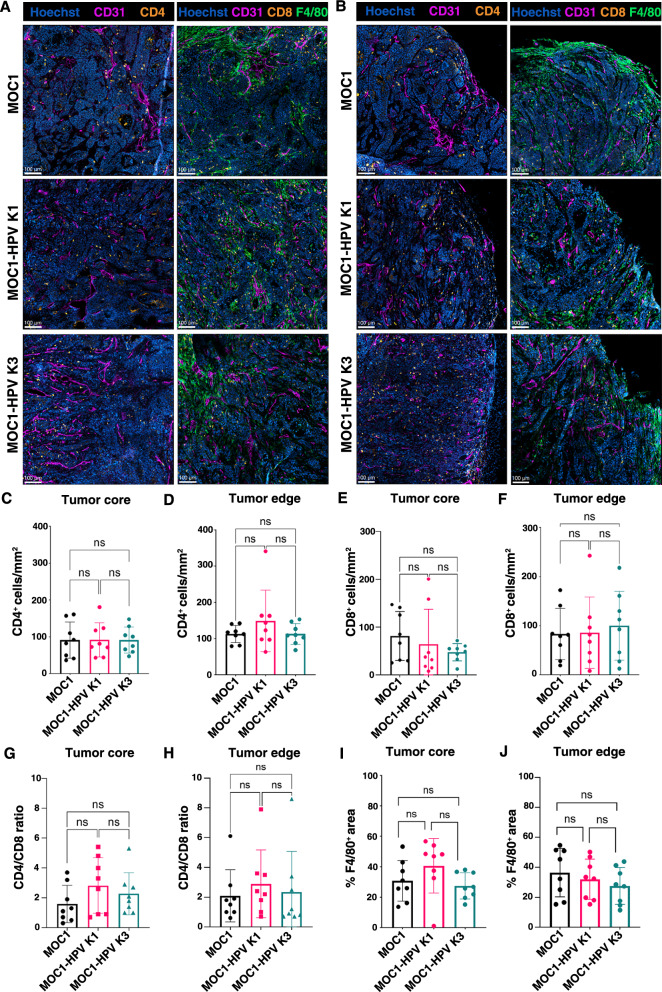
Immune cell infiltration of MOC1 and MOC1-HPV tumors. **A**, **B** Representative images of immunofluorescently stained frozen sections of the tumor core (**A**) or tumor edge (**B**). T lymphocytes were marked with CD4 or CD8 (orange), macrophages with F4/80 (green), blood vessels with CD31 (magenta), and cell nuclei with Hoechst 33,342 (blue). Scale bar: 100 µm. **C**,** D** Numbers of CD4 T lymphocytes per mm^2^ of tumor core (**C**) and tumor edge (**D**). **E**, **F** Numbers of CD8 T lymphocytes per mm^2^ of tumor core (**E**) and edge (**F**). **G**, **H** CD4/CD8 ratio in the tumor core (**G**) and tumor edge (**H**). **I**, **J** Percentage of F4/80-positive area in the tumor core (**I**) and tumor edge (**J**). Bar plots present the mean ± SD. n = 4. *ns* not significant, ordinary one-way ANOVA

Since there are reports that HPV-positive OSCC tumors have higher infiltration of immune cells than HPV-negative tumors [42, 43], we were also interested if E6 and E7 expression induced changes in immune cell infiltration in our tumor models. Therefore, we immunofluorescently stained for CD8 T lymphocytes, CD4 T lymphocytes, macrophages expressing the surface marker F4/80 and blood vessels expressing CD31 (Fig. 7A, B). A comparable number of CD4 T lymphocytes per mm^2^ of tumor area was detected in the tumor core (Fig. 7C) as well as in the tumor edge (Fig. 7D) in all three tumor models. A similar trend was observed for CD8 T lymphocytes (Fig. 7E, F). Although the average CD4/CD8 ratio was slightly higher in MOC1-HPV K1 and MOC1-HPV K3 tumors, the differences between the three tumor models were not statistically significant (Fig. 7G, H). Similarly, macrophages were present at comparable frequencies in all three tumor models in both the tumor core (Fig. 7I) and tumor edge (Fig. 7J).

### Response of MOC1 and MOC1-HPV cell lines and tumors to radiotherapy

**Fig. 8:**
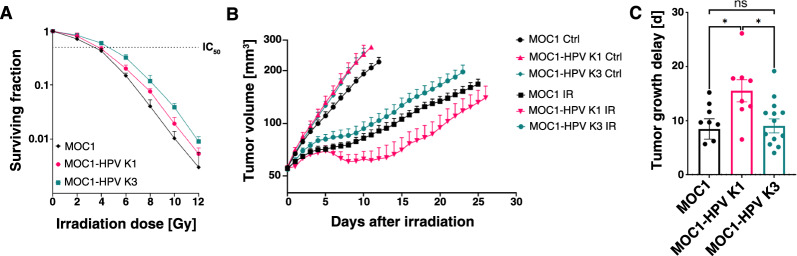
Radiosensitivity of MOC1, MOC1-HPV K1, and MOC1-HPV K3. **A** Radiosensitivity of MOC1 and MOC1-HPV cell lines as assessed with a clonogenic assay. Presented are the mean + SD. N = 3. **B** Tumor growth after irradiation with a single dose of 15 Gy. Presented are the mean + SEM. n = 9–14. **C** Tumor growth delay after irradiation with a single dose of 15 Gy. Presented are the mean ± SEM. n = 9–14. *ns* not significant, *p < 0.05, ordinary one-way ANOVA. *Ctrl* control, *IR* irradiated

Clinical reports suggest that patients with HPV-positive OSCC respond better to radiotherapy [14, 15]; therefore, we were interested in the effect of E6 and E7 expression on the radiosensitivity of MOC1-HPV cells. First, we determined the radiosensitivity of the three cell lines in vitro using a clonogenic assay. The reproductive integrity after irradiation with a single irradiation dose between 0 and 12 Gy did not differ significantly among all tested cell lines, resulting in comparable IC_50_ values calculated with the linear quadratic model—6.0 ± 1.3 Gy for MOC1, 5.8 ± 2.2 Gy for MOC1-HPV K1, and 4.9 ± 0.5 Gy for MOC1-HPV K3, as well as IC_90_ values of 9.9 ± 1.5 Gy for MOC1, 9.5 ± 2.5 Gy for MOC1-HPV K1, and 9.1 ± 0.7 Gy for MOC1-HPV K3 (Fig. 8A). Next, we examined the sensitivity of the subcutaneous tumor models to irradiation with a single dose of 15 Gy. By measuring tumor volumes after irradiation, we showed that irradiation with a single dose of 15 Gy slowed the growth of all three tumor models compared to the unirradiated controls (Fig. 8B), but the growth delay was significantly longer in MOC1-HPV K1 tumors (15.5 days compared to 8.5 days in MOC1 and 9 days in MOC1-HPV K3) (Fig. 8C).

## Discussion

In this study, we developed a syngeneic transplantable mouse tumor model of HPV-positive OSCC suitable for immunological studies in immunocompetent C57Bl/6 mice. The MOC1-HPV K1 tumor model exhibits increased radiosensitivity in vivo, reduced hypoxia, and increased proliferation compared to its HPV-negative MOC1 counterpart. Tumors present with CD8 and CD4 T lymphocytes in the tumor core and edge, which is crucial for the efficacy of immunotherapies. RNA sequencing of the MOC1 and MOC1-HPV cell lines demonstrated transcriptomic differences in angiogenesis, cell migration and cell adhesion-related pathways, which are reflected in functional in vitro assays and in in vivo tumor model characteristics.

Mouse models are crucial tools for preclinical research. Early studies of HPV-induced carcinogenesis used immunocompetent FVB/N or C57Bl/6 mouse strains genetically engineered to express HPV-16 E6 and E7 oncogenes under the control of the human keratin 14 (K14) promoter to direct transgene expression into stratified squamous epithelium [44–48]. Additionally, mutant oncogenes such as the mutated c-H-Ras gene or carcinogen treatment were included to increase the primary tumor formation rate [48]. To direct the expression of E6 and E7 to the head and neck region, inducible transgenic mouse models were developed using the Cre recombinase system, in which the expression of E6 and E7 can be induced by local intralingual administration of tamoxifen or doxycycline [49–51], resulting in OSCC that recapitulates the histological and molecular characteristics of human HPV-positive OSCC [49]. However, genetically engineered mouse tumor models are time-consuming and have variable tumor formation rates and locations. Therefore, transplanted mouse tumor models are easier to handle in the context of 3Rs due to the reduced variability of the experiments. Most available transplanted mouse models of HPV-positive OSCC are patient-derived xenografts (PDXs) that are transplanted subcutaneously into immunocompromised mice. There are several reports on establishing PDXs of HPV-positive and HPV-negative head and neck SCC, which retain the tumor characteristics of the donor and in some cases recapitulate patient clinical responses [33–36, 52]. The main obstacles in developing HPV-positive PDX models are low engraftment rates and often the formation of Epstein‒Barr virus-positive lymphomas derived from cotransfer of stroma from the tonsil or base of the tongue [34, 52], both of which contribute to higher costs. Moreover, because immunocompromised mouse strains are used for PDXs, their use in preclinical immunological studies is limited.

Because of the high tumor induction success rate and time effectiveness of transplantable immunocompetent mouse models, we aimed to develop an HPV-positive murine cell line of OSCC that would resemble the clinical features of HPV-positive OSCC and would enable both in vitro studies and in vivo studies in immunocompetent mice. Since HPV can only infect human cells, there are very few murine cell lines of HPV-positive OSCC. Hoover et al. and Williams et al. reported the establishment of an HPV-positive cell line, mEER, by transducing mouse pharyngeal epithelial cells isolated from C57Bl/6 mice with a retroviral vector encoding the H-*ras* and HPV-16 E6 and E7 oncogenes, capable of forming transplantable tumors [53, 54]. Similarly, MOC2-E6E7 cells were established by transduction of highly metastatic murine OSCC MOC2 cells with a retrovirus encoding HPV-16 E6 and E7 [55]. Furthermore, Paolini et al. established an HPV-positive cell line, AT-84-E7, by stably transfecting the AT-84 cell line with a plasmid containing the HPV-16 E7 oncogene. AT-84-E7 cells can be injected into the floor of the mouth of immunocompetent C3H mice to obtain an orthotopic mouse model of HPV-positive OSCC [56]. The characteristics of all available HPV-positive murine cell lines are described in Table 1.

**Table 1:** Comparison of the newly established MOC1-HPV tumor models with the existing models of HPV-positive OSCC

	MOC1-HPV	mEER and mEERL [54, 69]	MOC2-E6E7 [55]	AT-84-E7 and AT-84-E7-Luc [56, 91]
Method of establishment	Retroviral transduction	Retroviral transduction	Retroviral transduction	Transfection
Parental cells	MOC1 cell line	Primary mouse pharyngeal cells	MOC2 cell line	AT-84 cell line
Oncogenes and reporter genes	HPV-16 E6 and E7	H-Ras, HPV-16 E6 and E7; mEERL firefly luciferase reporter gene	HPV-16 E6 and E7	HPV-16 E7; AT-84-E7-Luc firefly luciferase reporter gene
In vitro				
Confirmation of E6, E7 expression	qRT‒PCR and IF	/	qRT‒PCR	qRT‒PCR, western blot
Proliferation	Comparable population doubling times (PrestoBlue™ metabolic assay)	E6 and E7 immortalized pharyngeal cells (reaching more than 25 population doublings), shorter population doubling time in mEER cells	Doubling time of MOC2-E6E7 comparable to MOC2 (cell count and viability)	/
Migration	MOC1 and MOC1-HPV K1 comparable migration velocity, MOC1-HPV K3 significantly slower	/	/	/
Gene expression analysis in vitro	RNA-sequencing	/	/	/
Radiosensitivity	Comparable radiosensitivity (clonogenic assay, 0–12 Gy)	mEER more resistant than wildtype pharyngeal cells (clonogenic assay)	/	/
In vivo				
Location	Subcutaneous (flank), orthotopic not tested	Orthotopic (intraoral) and subcutaneous (flank)	Orthotopic (maxillary vestibule of the oral cavity)	Subcutaneous (flank) or orthotopic (tongue)
Source mouse strain	C57Bl/6Ncrl	C57Bl/6	C57Bl/6J	C3H
Confirmation of E6, E7 expression	Determined at tumor volume 50 mm^3^ (qRT‒PCR)	Maintains expression with tumor growth (qRT‒PCR)[55]	Reduced E6 and E7 expression with tumor growth (qRT‒PCR)	Stable E7 expression maintained (qRT‒PCR)
Tumor inoculation	1 × 10^6^ cells (flank)	5 × 10^5^ cells (tongue), 1 × 10^6^ cells (flank)	3 × 10^4^ cells	6 × 10^5^ cells (floor of the mouth), 1 × 10^5^ cells (flank); forms lung metastases
Tumor formation rate	88% in both HPV models (flank)	Orthotopic 83.3%, subcutaneous 80%	/	/
HE	Well differentiated SCC, MOC1-HPV K1 more immune cell infiltration; keratin pearls present	Poorly differentiated SCC	Abundant lymphocyte infiltration	Appearance of sarcomatoid carcinoma or spindle cell squamous carcinoma, no keratinization
Tumor growth kinetics	Time to grow from 50–60 mm^3^ to 100 mm^3^ comparable in all three tumor models	Logarithmic growth	Significantly slower growth rate of MOC2-E6E7 in oral cavity compared to MOC2 in C57Bl/6J WT mice, but similar in Rag1^−/−^ mice	/
Tumor microenvironment	Differences in hypoxia and percentage of proliferating cells, blood vessel area comparable (immunofluorescence staining)	/	/	/
Immune profile	Comparable infiltration of CD4 and CD8 T lymphocytes in all tumor models (immunofluorescence staining). Trend toward higher CD4/CD8 ratio in MOC1-HPV K1 tumor model	/	T cell inflamed phenotype (nanoString); significantly more CD8 + TILs in MOC2E6E7 tumors compared to MOC2; CD4 + TILs similar in both (flow cytometry)	Approximately 1.5% of CD3^+^ cells are CD8^+^; approximately 1% of CD3^+^ cells are CD4^+^ (flow cytometry) [91]
Gene expression analysis in vivo	/	/	nCounter PanCancer Immune Profiling Panel (NanoString, WA, USA)	/
Radiosensitivity	MOC1-HPV K1 significantly longer growth delay after irradiation with a single dose of 15 Gy	80% of mice with mEER cells complete response after < 20 Gy irradiation [69]	/	Determined only in combination with immune checkpoint inhibitors (anti-PD-L1) [91]

Expression of the HPV oncogenes E6 and E7 can lead to several phenotypic changes in HPV-positive cells. Studies show that HPV oncogenes increase migration capacity in some cell types, including cervical cancer cells and trophoblastic cells [57–59], while the results of studies on HPV-positive head and neck SCC cells are inconclusive. Nagel et al. tested the migratory capacity of several OSCC cell lines using a wound-healing assay and showed that the migratory capacity of HPV-positive cell lines varies and is not significantly different from that of HPV-negative SCC cell lines [60]. Similarly, Kahue et al. showed that the migratory capacity of HPV-positive OSCC cell lines does not significantly differ from the migratory capacity of HPV-negative OSCC cell lines; however, when the expression of E6 and E7 is abrogated in HPV-positive cell lines, their migratory capacity decreases, indicating a potential role of E6 and E7 in migration properties [61]. In our study, differences in cell migration were detected both in in vitro functional assays and at the transcriptome level. However, the expression of HPV-16 E6 and E7 did not change the migration velocity in MOC1-HPV K1 cells and even decreased the migration velocity of MOC1-HPV K3 cells in comparison to the parental MOC1 cell line. Therefore, it seems that the effect on migration properties is multifactorial and cannot be attributed solely to HPV oncogenes. Furthermore, E6 and E7 impair cell adhesion in some cell types proposedly through E6-mediated downmodulation of focal adhesion [59, 62], which may contribute to the observed differences in migration and adhesion properties of the MOC1-HPV K1-cell line compared to the parental MOC1 cell line.

Studies on patient tumor samples observed differences in the tumor microenvironment of HPV-positive and HPV-negative tumors, namely, greater T lymphocyte infiltration with a lower CD4/CD8 ratio in HPV-positive OSCC tumors, which correlated with better overall survival [42, 43, 63–65]. Greater infiltration of CD8 T lymphocytes was also observed in flow cytometric evaluation of the orthotopic MOC2-E6E7 mouse tumor model [55] but not in the immunofluorescence staining of our subcutaneous MOC1-HPV tumor models, even though MOC1-HPV K1 tumors presented with higher lymphocyte and neutrophilic granulocyte infiltration on HE-stained paraffin-embedded tumor tissue sections. Therefore, the focus of further studies will be the precise characterization of the immune profile of MOC1-HPV tumors with flow cytometry, where multiple immune cell populations can be detected simultaneously in the whole tumor. Moreover, additional parameters could be assessed to better evaluate the immune phenotype of MOC1-HPV tumors. A study by Mytilineos et al. [66] investigated and compared the levels of circulating cytokines in serum of patients with HPV-negative and HPV-positive OSCC, which would be interested to investigate and compare in MOC1-HPV mouse model as well. Nevertheless, we have shown that MOC1-HPV tumors have an immune-inflamed phenotype [67, 68] with T lymphocyte infiltration both in the tumor edge and in the tumor core, making them suitable mouse models for immunotherapy studies.

Clinical data show increased radiosensitivity of HPV-positive OSCC, leading to improved patient survival [14, 15]. Subcutaneous mouse tumors derived from E6E7/Ras pharyngeal cells showed a better response to irradiation with a single dose between 8 and 32 Gy than tumors derived from HPV-negative shPTPN13/Ras pharyngeal cells [69]. However, the results of in vitro studies are inconclusive. Some report on higher radiosensitivity of HPV-positive cell lines in clonogenic or cell viability assays [70–72], while others observed no significant differences [60] or even lower radiosensitivity compared with HPV-negative cell lines [69]. Similarly, in this study, all three cell lines showed comparable radiosensitivity in vitro, but one of the tumor models, MOC1-HPV K1, presented with significantly higher sensitivity to irradiation with a single dose of 15 Gy in vivo, indicating that the intrinsic radiosensitivity of the cells is not the main factor determining the response of these tumor models to radiotherapy. The irradiation dose of 15 Gy was selected as the dose that would not cure the tumors, which would enable us to perform the growth delay assay and compare the radiosensitivity of the tumor models without generating dose response curves for each tumor model. The latter would require a significantly higher number of animals [73] and would not provide significantly more information on the radiosensitivity of the models.

One of the factors compromising the response to irradiation is hypoxia, which reduces the amount of radiation-induced free radicals and DNA damage [74]. In line with this, we observed significantly less tumor hypoxia in the more radiosensitive MOC1-HPV K1 tumor model. Based on literature data, more than half of patients with head and neck cancer present with tumor hypoxia [75, 76], with no statistically significant differences in the level or distribution of hypoxia among HPV-negative and HPV-positive tumors [76–78]. Interestingly, differences in hypoxia correlated with in vitro transcriptional differences in the pathways related to blood vessel morphogenesis and angiogenesis. Moreover, despite all three tumor models showing a comparable percentage of tumor blood vessel area, vessels in MOC1-HPV K1 tumors were significantly smaller, which could lead to better perfusion of these tumors [79]. Additional experiments to determine whether the functionality of the vessels in the three tumor models differ would therefore be of interest.

Differences in the characteristics of MOC1-HPV K1 and MOC1-HPV K3 tumor models could also be due to the different E6 and E7 integration sites in the established monoclonal cell lines. During persistent HPV infection, parts of the viral genome, including the E6 and E7 oncogenes, frequently integrate into the host genome. Based on currently available studies, HPV integration into the genome is random; however, more integration sites seem to be located in or in close proximity to transcriptionally active and genic regions [26, 80, 81]. In addition, a recent study by Mima et al. [38] suggests that epigenetic regulation of genes in the proximity of HPV integration sites may play an important role in the biology of HPV-related OSCC. Parfenov et al. reported on integration sites resulting in disruption of the DNA repair gene RAD51 homolog 2 (RAD51B) or tumor suppressor gene ETS2, elevated expression of the tumor oncogene NR4A2 or elevated expression of a PD-L1 splicing form, which could all contribute to HPV-driven carcinogenesis [26]. Therefore, determination of E6 and E7 integration sites in the monoclonal cell lines MOC1-HPV K1 and MOC1-HPV K3 through whole-genome sequencing would offer insight into the genomic changes induced by E6 and E7 retroviral integration and whether they are similar to those after HPV infection in patients.

This study and the developed HPV-positive mouse OSCC models have some limitations. First, the anatomical origin of the parental MOC1 cell line is not the oropharynx, which is clinically associated with better prognosis and increased radiosensitivity in patients [11, 13–15]. However, mice do not have the tonsillar crypt epithelium [82, 83] where most human HPV-positive oropharyngeal tumors arise [84]. Moreover, tumors in the study were not grown orthotopically, but rather a flank model was used due to the ease of tumor induction, measurement, handling, and therapy application. Furthermore, the MOC1 cell line originates from carcinogen-induced tumors and therefore presents with different driver mutations than HPV-positive OSCC tumors in patients. For example, MOC1 cells present with mutated p53, which is an important target of HPV oncogenes in HPV-induced carcinogenesis [85, 86]. Nevertheless, the developed MOC1-HPV K1 tumor model still presents with increased radiosensitivity similar to that observed in patients. To our knowledge, all available murine OSCC cell lines are derived from carcinogen-induced tumors, therefore we have chosen MOC1 for parental cell line since it has been extensively used in various studies of OSCC biology (reviewed in [87]). Another limitation of the study is that the transcriptomic analysis was performed only on the monoclonal cell lines in vitro. Transcriptomic differences at the in vitro level correspond well with the characteristics of the tumors induced in C57Bl/6 mice as well as with transcriptomic characteristics of human patient-derived OSCC cell lines. On the other hand, the most enriched GO Biological process pathways in MOC1-HPV cell lines as well as in patient-derived OSCC cell lines mainly do not overlap with those enriched in patient tumors. Therefore, transcriptomic profile characterization of MOC1-HPV tumors would be of interest for further studies in order to better determine the transcriptomic resemblance of MOC1-HPV tumors to HPV-positive OSCC tumors in patients. Moreover, to further increase the resemblance of MOC1-HPV models to patient tumors, different co-culture setups with naïve murine immune cells prior to tumor implantation could be investigated, similarly to the Winn Assay, where tumor and immune cells are mixed prior to tumor implantation [88–90]. This way, immune hot or immune cold MOC1-HPV tumors could potentially be established. Another promising approach for developing the MOC1-HPV model further would also be the co-culture of MOC1-HPV tumor cells with stromal or immune cells in 2D or 3D in vitro cell model. This could increase the complexity of the in vitro model and conform with the 3R animal welfare guidelines.

## Conclusions

Altogether, in this study, we established and systematically characterized two murine OSCC cell lines that stably express the HPV-16 oncogenes E6 and E7 and can be used for the induction of subcutaneous tumors in immunocompetent C57Bl/6 mice. One of the resulting mouse models of HPV-positive OSCC, MOC1-HPV K1, shows increased radiosensitivity similar to patient HPV-positive OSCC. To our knowledge, our developed syngeneic transplantable mouse tumor models are the best characterized tumor models described to date that allow us to study responses to immune-mediated treatment approaches in HPV-negative and HPV-positive OSCC, which is currently limited due to very few syngeneic HPV-positive murine OSCC cell lines.

## Supplementary Information

**Additional file 1: Figure S1**. MOC1-HPV cell lines show stable overall gene expression with cell culturing. Heatmap of differentially expressed genes with hierarchical clustering shows stable expression of differentially expressed genes between cells in passage 10 and passage 15. **Figure S2**. Cell migration kinetics of MOC1-HPV cell lines after extensive passaging. Cell front velocity of MOC1-HPV cells in the wound-healing assay after passaging for minimally 17 passages. Presented are mean ± SD. ***: p < 0.001, one-way ANOVA. **Table S1**. Most enriched GO Biological process pathways when comparing UM-SCC-104 (HPV-positive) and FaDu (HPV-negative) cell lines. Gene ontology analysis of differentially expressed genes. Pathways with an adjusted p value below 0.05 were considered to be enriched. **Table S2.** Most enriched GO Biological process pathways when comparing UM-SCC-47 (HPV-positive) and FaDu (HPV-negative) cell lines. Gene ontology analysis of differentially expressed genes. Pathways with an adjusted p value below 0.05 were considered to be enriched. **Table S3.** Most enriched GO Biological process pathways when comparing UM-SCC-090 (HPV-positive) and FaDu (HPV-negative) cell lines. Gene ontology analysis of differentially expressed genes. Pathways with an adjusted p value below 0.05 were considered to be enriched. **Table S4**. Most enriched GO Biological process pathways when comparing RNA-sequencing data from HPV-positive and HPV-negative patient tumor samples. Gene ontology analysis of differentially expressed genes. Pathways with an adjusted p value below 0.05 were considered to be enriched.

**Additional file 2:** Supplementary methods.

## Data Availability

The data that support the findings of this study are available from the corresponding authors upon reasonable request. RNA-sequencing data obtained and discussed in this publication have been deposited to the NCBI Gene Expression Omnibus (GEO) and are accessible through GEO Series accession number GSE224448 (https://www.ncbi.nlm.nih.gov/geo/query/acc.cgi?acc=GSE224448).

## Abbreviations

OSCC: Oral squamous cell carcinoma
HPV: Human papillomavirus
pRb: Retinoblastoma protein
mRNA: Messenger ribonucleic acid
qRT-PCR: Quantitative real-time polymerase chain reaction
HE: Hematoxylin and eosin
GO: Gene ontology
PDX: Patient-derived xenograft
SCC: Squamous cell carcinoma
IC50: Half maximal inhibitory concentration
IMDM: Iscove’s Modified Dulbecco’s Medium
FBS: Fetal bovine serum
EGF: Epidermal growth factor
padj: Adjusted p value
