# Supplementary material for: HPV-positive murine oral squamous cell carcinoma: development and characterization of a new mouse tumor model for immunological studies
Source: J Transl Med. 2023 Jun 10;21:376. doi: 10.1186/s12967-023-04221-4 (PMC10257320; doi:10.1186/s12967-023-04221-4)
Supplement: Supplementary file 1 — Additional file 1: Figure S1. MOC1-HPV cell lines show stable overall gene expression with cell culturing. Heatmap of differentially expressed genes with hierarchical clustering shows stable expression of differentially expressed genes between cells in passage 10 and passage 15. Figure S2. Cell migration kinetics of MOC1-HPV cell lines after extensive passaging. Cell front velocity of MOC1-HPV cells in the wound-healing assay after passaging for minimally 17 passages. Presented are mean ± SD. ***: p < 0.001, one-way ANOVA. Table S1. Most enriched GO Biological process pathways when comparing UM-SCC-104 (HPV-positive) and FaDu (HPV-negative) cell lines. Gene ontology analysis of differentially expressed genes. Pathways with an adjusted p value below 0.05 were considered to be enriched. Table S2. Most enriched GO Biological process pathways when comparing UM-SCC-47 (HPV-positive) and FaDu (HPV-negative) cell lines. Gene ontology analysis of differentially expressed genes. Pathways with an adjusted p value below 0.05 were considered to be enriched. Table S3. Most enriched GO Biological process pathways when comparing UM-SCC-090 (HPV-positive) and FaDu (HPV-negative) cell lines. Gene ontology analysis of differentially expressed genes. Pathways with an adjusted p value below 0.05 were considered to be enriched. Table S4. Most enriched GO Biological process pathways when comparing RNA-sequencing data from HPV-positive and HPV-negative patient tumor samples. Gene ontology analysis of differentially expressed genes. Pathways with an adjusted p value below 0.05 were considered to be enriched. [file 12967_2023_4221_MOESM1_ESM.docx]

**Additional file 1: Supplementary Figures and Tables**


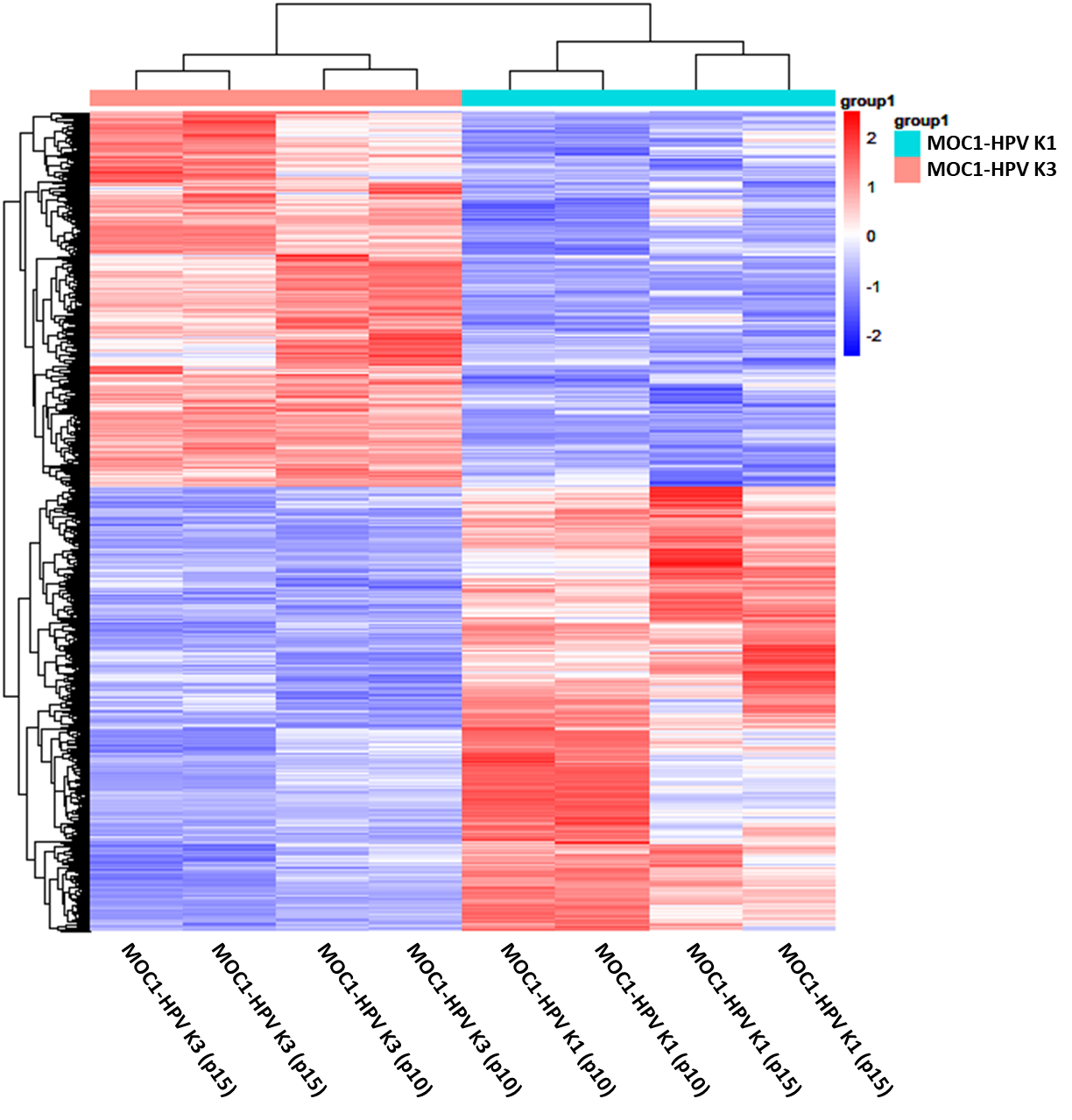


**Figure S1: MOC1-HPV cell lines show stable overall gene expression with cell culturing.** Heatmap of differentially expressed genes with hierarchical clustering shows stable expression of differentially expressed genes between cells in passage 10 and passage 15.


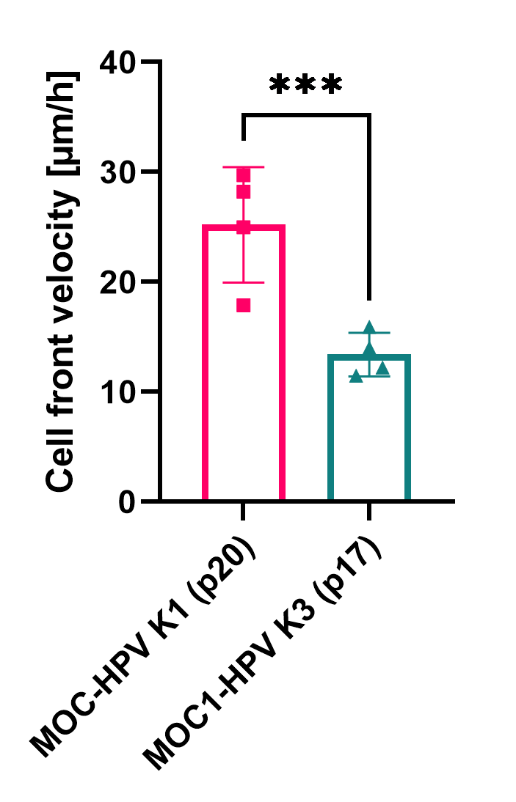


**Figure S2: Cell migration kinetics of MOC1-HPV cell lines after extensive passaging.** Cell front velocity of MOC1-HPV cells in the wound-healing assay after passaging for minimally 17 passages. Presented are mean ± SD. ***: p < 0.001, one-way ANOVA.

**Table S1: Most enriched GO Biological process pathways when comparing UM-SCC-104 (HPV-positive) and FaDu (HPV-negative) cell lines.** Gene ontology analysis of differentially expressed genes. Pathways with an adjusted p value below 0.05 were considered to be enriched.

| Pathways | adj.Pval |
| --- | --- |
| Extracellular matrix organization | 9.50E-09 |
| Extracellular structure organization | 9.50E-09 |
| External encapsulating structure organization | 9.50E-09 |
| Anatomical structure morphogenesis | 4.78E-08 |
| Negative regulation of intracellular signal transduction | 6.34E-08 |
| Blood vessel development | 1.14E-07 |
| Negative regulation of signal transduction | 1.15E-07 |
| Tube development | 1.15E-07 |
| Negative regulation of response to stimulus | 1.15E-07 |
| Cell migration | 1.29E-07 |
| Vasculature development | 1.83E-07 |
| Negative regulation of cell communication | 1.83E-07 |
| Negative regulation of signaling | 1.83E-07 |
| Locomotion | 1.83E-07 |
| Tube morphogenesis | 2.11E-07 |
| Type I interferon signaling pathway | 9.04E-04 |
| Cellular response to type I interferon | 9.04E-04 |
| MRNA metabolic process | 1.63E-03 |
| Heterocycle biosynthetic process | 1.63E-03 |
| Nucleobase-containing compound biosynthetic process | 1.81E-03 |

**Table S2:** **Most enriched GO Biological process pathways when comparing UM-SCC-47 (HPV-positive) and FaDu (HPV-negative) cell lines.** Gene ontology analysis of differentially expressed genes. Pathways with an adjusted p value below 0.05 were considered to be enriched.

| Pathways | adj.Pval |
| --- | --- |
| Extracellular matrix organization | 2.96E-14 |
| Extracellular structure organization | 2.96E-14 |
| External encapsulating structure organization | 2.97E-14 |
| Anatomical structure morphogenesis | 4.74E-10 |
| Cell migration | 4.74E-10 |
| Tube morphogenesis | 7.75E-09 |
| Tissue development | 8.87E-09 |
| Cell motility | 8.87E-09 |
| Localization of cell | 8.87E-09 |
| Regulation of anatomical structure morphogenesis | 1.13E-08 |
| Locomotion | 1.13E-08 |
| Tube development | 1.25E-08 |
| Blood vessel development | 1.62E-08 |
| Cell adhesion | 1.62E-08 |
| Biological adhesion | 1.62E-08 |
| Nuclear-transcribed mRNA catabolic process, nonsense-mediated decay | 1.55E-04 |
| Nuclear-transcribed mRNA catabolic process | 1.55E-04 |
| Cytoplasmic translation | 1.55E-04 |
| Cotranslational protein targeting to membrane | 1.55E-04 |
| SRP-dependent cotranslational protein targeting to membrane | 1.55E-04 |

**Table S3:** **Most enriched GO Biological process pathways when comparing UM-SCC-090 (HPV-positive) and FaDu (HPV-negative) cell lines.** Gene ontology analysis of differentially expressed genes. Pathways with an adjusted p value below 0.05 were considered to be enriched.

| Pathways | adj.Pval |
| --- | --- |
| Translation | 2.25E-38 |
| Peptide biosynthetic process | 4.17E-38 |
| Amide biosynthetic process | 4.48E-37 |
| Peptide metabolic process | 1.67E-36 |
| Cytoplasmic translation | 1.75E-36 |
| Cellular amide metabolic process | 1.00E-35 |
| Translational initiation | 1.79E-33 |
| Ribosome biogenesis | 1.53E-32 |
| SRP-dependent cotranslational protein targeting to membrane | 2.28E-32 |
| Ribonucleoprotein complex biogenesis | 6.38E-32 |
| Protein targeting to ER | 2.96E-30 |
| Organonitrogen compound biosynthetic process | 2.70E-29 |
| Establishment of protein localization to endoplasmic reticulum | 7.94E-29 |
| RRNA processing | 2.21E-27 |
| Nuclear-transcribed mRNA catabolic process, nonsense-mediated decay | 1.88E-26 |
| Nervous system development | 3.94E-24 |
| Anatomical structure morphogenesis | 5.59E-21 |
| Neurogenesis | 2.62E-20 |
| Generation of neurons | 4.27E-19 |
| Neuron differentiation | 3.40E-18 |
| Regulation of developmental process | 6.68E-17 |

**Table S4:** **Most enriched GO Biological process pathways when comparing RNA-sequencing data from HPV-positive and HPV-negative patient tumor samples.** Gene ontology analysis of differentially expressed genes. Pathways with an adjusted p value below 0.05 were considered to be enriched.

| Pathways | adj.Pval |
| --- | --- |
| SRP-dependent cotranslational protein targeting to membrane | 3.31E-69 |
| Protein targeting to ER | 1.02E-65 |
| Establishment of protein localization to endoplasmic reticulum | 2.47E-65 |
| Protein localization to endoplasmic reticulum | 7.64E-59 |
| Cytoplasmic translation | 2.69E-56 |
| Peptide metabolic process | 6.40E-53 |
| Nuclear-transcribed mRNA catabolic process, nonsense-mediated decay | 1.87E-52 |
| Translation | 4.34E-52 |
| Translational initiation | 5.84E-52 |
| Peptide biosynthetic process | 1.84E-51 |
| Amide biosynthetic process | 6.63E-46 |
| Protein targeting to membrane | 1.15E-44 |
| Cellular amide metabolic process | 1.32E-43 |
| Viral transcription | 5.48E-41 |
| Nuclear-transcribed mRNA catabolic process | 9.16E-39 |
| Regulation of nucleobase-containing compound metabolic process | 2.25E-15 |
| Regulation of transcription, DNA-templated | 4.52E-15 |
| Regulation of nucleic acid-templated transcription | 4.52E-15 |
| Regulation of RNA biosynthetic process | 4.52E-15 |
| Chromosome organization | 6.03E-15 |
