# Supplementary material for: HPV-positive murine oral squamous cell carcinoma: development and characterization of a new mouse tumor model for immunological studies
Source: J Transl Med. 2023 Jun 10;21:376. doi: 10.1186/s12967-023-04221-4 (PMC10257320; doi:10.1186/s12967-023-04221-4)
Supplement: Supplementary file 2 — Additional file 2: Supplementary methods. [file 12967_2023_4221_MOESM2_ESM.docx]

# Additional file 2: Supplementary methods

## Cell lines

The murine OSCC cell line MOC1 was purchased from Kerafast (Boston, MA, USA). Cells were cultured according to the manufacturer’s instructions in MOC1 cell line medium, consisting of Iscove's Modified Dulbecco's Medium (IMDM)/Ham’s F12 Nutrient Mixture (Gibco, Thermo Fisher Scientific, Waltham, MA, USA), in a ratio of 2:1, supplemented with 5% (v/v) fetal bovine serum (FBS, Gibco), 100 U/mL penicillin, 100 µg/mL streptomycin (100x penicillin‒streptomycin, Sigma Aldrich, Darmstadt, Germany), 5 µg/mL insulin (Sigma Aldrich), 40 ng/mL hydrocortisone (Sigma Aldrich) and 5 ng/mL epidermal growth factor (EGF, Gibco).

The packaging cell line PA317 LXSN 16E6E7 (ATCC® CRL-2203) was purchased from American Type Culture Collection (ATCC, Manassas, VA, USA) and cultured in Dulbecco's Modified Eagle's Medium (DMEM) supplemented with 10% (v/v) FBS, 100 U/mL penicillin, 100 µg/mL streptomycin and 2 mM GlutaMAX (Gibco).

Cells were maintained at 37 °C in a 5% CO_2_ humidified atmosphere. Cells were subcultured twice weekly by detachment with 0.25% trypsin-EDTA solution (Gibco). MOC1 cells were routinely tested for mycoplasma infection using the MycoAlert^TM^ PLUS Mycoplasma Detection Kit (Lonza, Basel, Switzerland) and were mycoplasma-free.

## Establishment of MOC1-HPV cell lines

The PA317 LXSN 16E6E7 packaging cell line was used to produce amphotropic retroviral particles containing the human papilloma virus (HPV) type 16 E6 and E7 genes and a gene for neomycin resistance. To this end, 1×10^5^ PA317 LXSN 16E6E7 cells were seeded into T25 flasks in 4 mL of medium. After 24 h, the medium containing the retroviral particles was collected and filtered through a 0.45-µm filter. The virus-containing filtrate was stored at 4 °C until further use.

For transduction, 2×10^5^ MOC1 cells were seeded into wells of 6-well plates. After 24 h, the cell medium was removed and replaced with 1 mL of the virus-containing filtrate and 1 mL of fresh medium containing 4 µg/mL polybrene (Merck Millipore, Darmstadt, Germany). Following an 18-h incubation at 37 °C, the medium was removed, the cells were washed three times with PBS, and fresh medium was added. The next day, the cells were washed three times with PBS, and the medium was replaced with the selection medium (MOC1 cell line medium containing 600 µg/mL G418 disulfate (AppliChem, Darmstadt, Germany)). Cells were cultured in the selection medium for eight days, reseeding them to larger culture flasks when needed. The cell medium was exchanged with fresh selection medium every three days. Afterward, the cells were maintained in MOC1-HPV cell line medium (MOC1 cell line medium containing 200 µg/mL G418 disulfate).

To establish pure monoclonal cell lines, the pool of surviving cells after G418 disulfate selection was serially diluted to a concentration of 9 cells/mL, and 100 µL of the cell suspension was seeded into each well of a 96-well plate. Wells containing only a single cell that successfully formed colonies and replicated to confluency were reseeded into T75 cell culture flasks for further culturing. Two of the established monoclonal cell lines used in this study were named MOC1-HPV K1 and MOC1-HPV K3.

MOC1-HPV and PA317 LXSN 16E6E7 cell lines are classified as biosafety level 2, for which we acquired permission nr. 35421-6/2021-2550-4 from the Scientific committee for work with genetically modified organisms in contained use from the Government of the Republic of Slovenia.

## RNA isolation, reverse transcription and quantitative real-time polymerase chain reaction (qRT‒PCR)

To confirm stable expression of the HPV16 E6 and E7 oncogenes, qRT‒PCR for E6 and E7 was performed on both MOC1-HPV cell lines at cell passages 10, 15, and 20. RNA was extracted using the peqGOLD Total RNA Kit (VWR, West Chester, PA, USA) following the manufacturer’s instructions. The concentration of the isolated RNA and its quality were determined spectrophotometrically using a Cytation 1 Cell Imaging Multi-Mode Reader (Biotek, Winooski, VT, USA) by measuring the absorbance at 260 nm (A_260_). The purity was assessed by the A_260_/A_280_ ratio.

Reverse transcription of 1000 ng of total RNA into cDNA was performed using the SuperScript VILO cDNA Synthesis Kit (Invitrogen, Thermo Fisher Scientific) according to the manufacturer’s instructions. For qRT‒PCR, 10 ng of cDNA was mixed with PowerUp™ SYBR™ Green Master Mix and a set of forward and reverse primers for E6 (F-GACCCAGAAAGTTACCACAGTT, R-GTTGCTTGCAGTACACACATTC), E7 (F-TCAGAGGAGGAGGATGAAATAGA, R-GCACAACCGAAGCGTAGA), GAPDH (F-TTCACCACCATGGAGAAGGC, R-GGCATGGACTGTGGTCATGA), or β-actin genes (F-GAAGTGTGACGTTGACATCC, R-ACTCATCGTACTCCTGCTTG) in a final reaction volume of 20 µL. The reactions were run in duplicate on a QuantStudio™ 3 Real-Time PCR System. The cycling conditions were as follows: 2 min at 50 °C, 2 min denaturation at 95 °C, 40 cycles of 15 s at 95 °C and 1 min at 60 °C, and for melting curve determination, 15 s at 95 °C, 1 min at 60 °C, and 15 s at 95 °C. The analysis was performed by normalizing the expression of genes of interest (E6 and E7) to the average of the housekeeping genes (GAPDH and β-actin) using the ΔCt method. A gene was annotated as nondetermined (N.D.) when the Ct value was above the 35^th^ cycle.

## Immunofluorescence staining of cytospins

To confirm the expression of E6 and E7 in MOC1-HPV cell lines at the protein level, immunofluorescence staining of cytospins was used. Cytospins were prepared using a cytocentrifuge (Thermo Scientific Shandon Cytospin R 4 Cytocentrifuge, Waltham, MA, USA) by centrifugation at 700 rpm for 4 min at room temperature (RT), followed by methanol fixation. Cytospins were then stained with primary and secondary antibodies in a humidified, light-protected chamber. Briefly, cytospins were washed in PBS and permeabilized and blocked in block/perm buffer (PBS with 5% donkey serum (VWR), 0.5% Triton X100 (Thermo Fisher Scientific), and 22.52 mg/mL glycine (Sigma Aldrich)) for 30 min. Then, cytospins were washed and blocked in blocking buffer (PBS with 2% donkey serum and 22.52 mg/mL glycine) for 1 h. Samples were incubated with E6 or E7 primary antibodies (rabbit anti-HPV-16 E6, rabbit anti-HPV-18 E7, Thermo Fisher Scientific) diluted 1:200 in blocking buffer overnight at 4 °C. The next day, cytospins were washed three times with PBS and incubated with secondary antibody (donkey-anti rabbit Alexa Fluor 488, Jackson ImmunoResearch Labs, PA, USA) diluted 1:500 in PBS for 1 h at RT. Next, slides were washed three times in PBS and incubated with Hoechst 33342 solution (3 µg/mL in PBS, Thermo Fisher Scientific) for 10 min. After washing again three times with PBS, cytospins were mounted with Prolong Glass Antifade Mountant (Thermo Fisher Scientific). Immunofluorescently stained cytospins were imaged using an LSM800 confocal microscope (Carl Zeiss, Oberkochen, Germany) under a 63x objective (NA 1.4) using lasers with excitation wavelengths of 405 nm and 488 nm for Hoechst and Alexa Fluor 488, respectively. Emitted light was captured with a gallium arsenide phosphide (GaAsP) detector through variable dichroic and filters at channel-specific wavelengths: 410–545 nm for Hoechst 33342 and 488–545 nm for Alexa Fluor 488. Images were analyzed with Imaris software (Oxford Instruments, Oxford, United Kingdom).

## Cell morphology

To better observe the morphology of the attached cells, the cell membrane was stained with wheat germ agglutinin (WGA, Thermo Fisher Scientific), and nuclei were counterstained with Hoechst 33342 solution. Briefly, the day before staining, 1×10^4^ cells were seeded into each well of a 12-well chamber slide (Ibidi, Gräfelfing, Germany). The next day, the cells were washed in HBSS containing Ca^2+^ and Mg^2+^ for 5 min at RT. Then, cells were fixed with 200 µL 4% paraformaldehyde (PFA; VWR) for 15 min at 37 °C and washed three times in 200 µL of HBSS for 5 min at RT. To label the cell membranes, 200 µL of 1 µg/mL Alexa Fluor 647-labeled WGA solution was added to the cells for 10 min at RT. After incubation, the cells were washed twice with HBSS, and nuclei were counterstained with 3 µg/mL Hoechst 33342 pentahydrate for 10 min at RT. After washing with PBS, slides were mounted using ProLong™ Glass Antifade Mountant. Slides were imaged using an LSM800 confocal microscope under a 20x objective (NA 0.8). Hoechst 33342 and Alexa Fluor 647 were excited using lasers with excitation wavelengths of 405 nm and 640 nm, respectively. Emitted light was captured with a GaAsP detector through a variable dichroic and filters at channel-specific wavelengths: 410–545 nm for Hoechst 33342 and 645–700 nm for Alexa Fluor 647.

To determine the size of adherent cells, the largest diameter of every cell was measured on immunofluorescence (IF) images using the Fiji image processing package of ImageJ [1]. To assess the size of cells in suspension, cells were trypsinized, centrifuged and resuspended in MOC1 medium. The size of the cells was determined using a CytoSMART cell counter (CytoSMART Technologies B.V., Eindhoven, The Netherlands).

## Cell proliferation assay

To determine the proliferation kinetics of the cell lines, 500 MOC1, MOC1-HPV K1, or MOC1-HPV K3 cells in 100 µL of MOC1 cell line medium were seeded per well of a 96-well plate and allowed to attach for 3 h. Afterward, 10 µL of PrestoBlue™ Cell Viability Reagent (Invitrogen) was added to the cells. After 90 min of incubation, the fluorescence intensity was measured on a Cytation 1 Cell Imaging Multi-Mode Reader using 530/25 excitation and 590/35 emission filters. The assay was repeated every 24 h until 96 h postseeding. The results from each time point were normalized to the first time point.

## Wound healing assay

To determine the migrational properties of the cell lines, the wound healing assay was performed using 24-well plates with two-well silicone inserts (Ibidi). A total of 2x10^4^ cells were seeded into each chamber of the insert and grown until confluent. Afterward, the inserts were removed to acquire a 500 µm ± 100 µm cell-free gap, and the wells were imaged every hour for 20 h using a Cytation 1 Cell Imaging Multi-Mode Reader. Wound confluency was calculated at every time point using Gen5 software (BioTek). The wound closure speed was determined from the slope of the linear part of the cell area curve, and then the cell front velocity was calculated as previously described [2] using the formula $\text{V}_{\text{mig}}\text{=}\frac{\text{slope}}{\text{2x wound length}}$.

## RNA sequencing and transcriptome analysis

For transcriptome analysis, total RNA was isolated from four cell passages ranging from p8 to p15 as described above, yielding four biological replicates per cell line. The purity of the isolated RNA was assessed spectrophotometrically using the A_260_/A_280_ and A_260_/A_230_ ratios with a Cytation 1 Cell Imaging Multi-Mode Reader. The quality of the RNA was assessed using the RNA Assay for LabChip® GX Touch (PerkinElmer, [Waltham, Massachusetts, ZDA](https://www.google.com/search?rlz=1C1GCEA_enSI1009SI1009&q=Waltham&stick=H4sIAAAAAAAAAOPgE-LUz9U3MMuNLzBS4gAxM6qMTbW0spOt9POL0hPzMqsSSzLz81A4VhmpiSmFpYlFJalFxYtY2cMTc0oyEnN3sDLuYmfiYAAAslROp1UAAAA&sa=X&ved=2ahUKEwisq9bmoen4AhWuVfEDHZ1lC3MQmxMoAXoECEsQAw)) according to the manufacturer’s instructions. Samples with an RNA Quality Score higher than 6.8 were sent for transcriptome sequencing to Novogene (UK) Company LTD (Cambridge, United Kingdom). mRNA sequencing was performed on the Illumina platform. Quality control of raw reads was performed using fastp, and cleaned paired-end reads were mapped to the reference genome using HISAT2 software. The FeatureCounts package was used to count the reads mapped to each gene, and reads per kilobase of exon model per million mapped reads (RPKM) were calculated. Differential expression analysis was performed using the DESeq2 R package. P values were adjusted using Benjamini and Hochberg’s approach to control for the false discovery rate (FDR). Genes with an adjusted p value < 0.05 and a fold change > 1 were considered differentially expressed. Gene Ontology (GO) enrichment analysis of differentially expressed genes was processed through the ClusterProfiler R package. GO terms with an adjusted p value < 0.05 were considered significantly enriched.

## Animals and tumor induction

Female C57BL/6NCrl mice (Charles River, Calco (Lecco), Italy) were housed at RT with a 12 h light-dark cycle and food and water ad libitum. Experiments were approved by the Ministry of Agriculture, Forestry and Food of the Republic of Slovenia (permission no. U34401-35/2020/8). Up to six mice were housed per cage in specific-pathogen free conditions in a carousel mouse IVC rack system (Animal Care Systems Inc., Revere Parkway, USA). Each cage was provided with enrichment (Bio-Serv, VWR).

Tumors were induced in 8- to 12-week-old mice by subcutaneously injecting 1×10^6^ MOC1, MOC1-HPV K1, or MOC1-HPV K3 cells resuspended in 100 µL of saline solution into the flank of each mouse. Mice were monitored daily for signs of distress and weighed three times a week. Furthermore, tumors were measured three times a week using a digital Vernier caliper. Tumor volume was calculated using the formula for ellipsoid (V = a × b × c × π/6, where a, b, and c are perpendicular diameters of the tumor).

## Tumor collection

When tumors reached volumes of 50-60 mm^3^, mice were intraperitoneally injected with 200 µL of 10 mg/mL EdU solution (5-ethynyl-2’-deoxyuridine; Abcam, Cambridge, United Kingdom) and 200 µL of 10 mM EF5 solution (EF5 Hypoxia Detection Kit, Cyanine 3; EMD Millipore, CA, USA), which mark proliferating cells and hypoxic areas, respectively. After three hours, the animals were sacrificed, and tumors were excised and cut in half. Each half of the tumor was used either for determining the expression of HPV-16 E6 and E7 or immunofluorescence staining of frozen tissue sections to assess the tumor microenvironment. For histological analysis, tumors were collected when they reached either 50-60 mm^3^ or 100 mm^3^.

Tumor halves for determination of HPV-16 E6 and E7 expression (n= 3–5) were snap-frozen in liquid nitrogen and stored at -80 °C. Frozen tumor samples were crushed with a pestle and stored at -80 °C until RNA extraction.

Tumor halves for immunofluorescence staining (n = 4) were fixed in 4% PFA for 12-16 h at 4 °C. Thereafter, tumors were dehydrated in 30% sucrose solution for 24 h at RT, embedded in optimal cutting temperature compound (Tissue-Tek O.C.T. compound, Sakura Finetek, VWR), snap-frozen on dry ice, and stored at -80 °C.

Tumors used for tumor histology assessment (n = 4) were fixed in 10% neutral buffered formalin overnight and then transferred into 70% ethanol until embedding in paraffin.

## Determination of HPV-16 E6 and E7 *in vivo*

Thirty milligrams of crushed tumor tissue was used for RNA extraction. TRI reagent (Thermo Fisher Scientific) was used for cell lysis. After chloroform incubation, the aqueous phase was used for RNA isolation using the peqGOLD Total RNA Kit following the manufacturers’ instructions. Reverse transcription and qRT‒PCR were performed as described above (see 2.3).

## Tumor histology and microenvironment assessment

Paraffin-embedded tumors were sectioned into 2-µm-thick sections and stained with hematoxylin and eosin (HE) according to standard procedures. Sections were imaged using a BX-51 microscope (Olympus, Düsseldorf, Germany). Slides were evaluated by an experienced pathologist.

Slides with 14-µm-thick tissue sections were prepared from frozen OCT samples using a Leica CM1850 cryostat. Three marker combinations were stained on two slides. One slide was stained for hypoxia (EF5), proliferation (EdU), and blood vessels (CD31). Another slide was stained for immune cells – CD4-positive T cells and blood vessels (CD31) on one section and CD8-positive T cells, macrophages (F4/80), and blood vessels (CD31) on another section.

Staining was performed in a light-protected, humidified chamber. Before staining, sections were air-dried for 10 min at 37 °C and washed with 1X PBS for 5 min. Slides stained for proliferation (EdU), hypoxia (EF5), and blood vessels (CD31) were blocked and permeabilized in block/perm buffer for 1 h at RT and washed twice in blocking buffer. To detect the incorporated EdU Click-iT™ EdU Cell Proliferation Kit for Imaging, Alexa Fluor™ 488 dye (Invitrogen) was used following the manufacturer’s instructions. Briefly, slides were incubated with Click-iT mix for 30 min at RT and then washed with blocking buffer. Afterward, slides were incubated overnight at 4 °C with the respective primary antibodies diluted 1:200 in blocking buffer (Table 1). The next day, slides were blocked for 30 min in blocking buffer at RT before incubation with anti-EF5 antibodies conjugated to Cy3 overnight at 4 °C. The next day, slides were washed with PBS-T (PBS with 0.3% Tween-20 (Sigma Aldrich)) twice for 45 min at RT. Nuclei were counterstained with 3 µg/mL Hoechst 33342 in PBS for 5 min at RT. After washing with PBS, the slides were mounted using ProLong™ Glass Antifade Mountant (Invitrogen). Slides stained for immune cells were stained similarly, with added antigen retrieval after the first PBS wash, by incubating the slides in sodium citrate buffer (10 mM sodium citrate, 0.05% Tween 20, pH 6.0) heated to 95 °C for 30 min at RT, followed by 30 min of cooling in RT water. Then, slides were blocked and permeabilized in block/perm buffer for 30 min at RT, washed with blocking buffer, and then blocked in blocking buffer for 1 h at RT. Staining with primary antibodies was performed overnight at 4 °C, followed the next day by 3 washes with PBS and incubation with secondary antibodies at RT for 1 h. After 3 PBS washes, nuclear counterstaining and mounting were performed as described above.

Immunofluorescently stained tumor sections were imaged using an LSM 800 confocal microscope (Carl Zeiss) using a 10× (NA 0.45) or 20× objective (NA 0.8). Hoechst 33342, Alexa Fluor 488, Cy3 and Alexa Fluor 647 were excited with lasers with excitation wavelengths of 405 nm, 488 nm, 561 nm and 640 nm, respectively. Emitted light was captured with a GaAsP detector through a variable dichroic and filters at channel-specific wavelengths: 410–545 nm for Hoechst 33342, 488–545 nm for Alexa Fluor 488, 565–620 nm for Cy3, and 645–700 nm for Alexa Fluor 647. On sections stained for hypoxia, proliferating cells and blood vessels, two representative 3×3 tile scans of the tumor core were imaged, while on sections stained for immune cell markers, two representative 3×3 tile scans of the tumor edge were imaged as well. Images were analyzed with Imaris software (Oxford Instruments).

Table 1: Primary and secondary antibodies used for immunofluorescence staining

| **Antibody** | **Supplier** | **Cat. No.** |
| --- | --- | --- |
| Polyclonal goat anti-CD31/PECAM-1 antibody | R&D Systems | AF3628 |
| Rabbit monoclonal anti-CD4 antibody, clone EPR19514 | Abcam | ab183685 |
| Rabbit monoclonal anti-CD8 antibody, clone EPR20305 | Abcam | ab209775 |
| Rat anti-F4/80 antibody, clone BM8 | Thermo Fisher Scientific | 14-4801-82 |
| Mouse anti-EF5 antibody (Cy3 conjugate), clone ELK3-51 | EMD Millipore | AF3628-SP, part no. CS222730 |
| Donkey anti-Rabbit IgG antibody (Cy3®) | Jackson Immunoresearch | 711-165-152 |
| Donkey anti-goat IgG antibody (Alexa Fluor® 647) | Jackson Immunoresearch | 705-605-147 |
| Donkey anti-rat IgG (Alexa Fluor® 488) | Jackson Immunoresearch | 712-545-150 |

## Radiosensitivity evaluation *in vitro* and *in vivo*

To assess the *in vitro* reproductive integrity of cells after irradiation, a clonogenic assay was performed [3]. Cells were trypsinized, counted, and seeded into wells of a 6-well plate according to the irradiation dose they received (control - 50, 2 Gy - 100, 4 Gy - 300, 6 Gy - 1000, 8 Gy - 3000, 10 Gy – 5000, 12 Gy - 8000 cells). After attachment, ~3 h after seeding, they were irradiated with the Gulmay 225 X-ray system (Gulmay Medical Ltd., Byfleet, UK) with 0.55 mm Cu and 1.8 mm Al filtering. The dose rate was 1.73 Gy/min. After irradiation, plates were incubated in a 5% CO_2_ humidified incubator at 37 °C for six days before the colonies were fixed and stained with crystal violet staining solution (0.5% crystal violet (Sigma Aldrich) in 80% methanol). Colonies consisting of at least 50 cells were counted. Plating efficiency was calculated as the ratio between the number of counted colonies and the number of seeded cells. The surviving fraction of the cells was determined by normalizing the plating efficiencies of the irradiated groups to the plating efficiency of the unirradiated control group.

To evaluate the radiosensitivity of tumors *in vivo,* mice were randomly divided into the control group (n = 11–12) and irradiated group (n = 9–14) when tumor volumes reached 50-60 mm^3^ using a GraphPad QuickCalcs random number generator. Mice assigned to the irradiated group were irradiated with a single dose of 15 Gy using the Gulmay 225 X-ray system with 0.55 mm Cu and 1.8 mm Al filtering, and 1.92 Gy/min dose rate. Local delivery of the irradiation dose to the tumor was ensured using lead shielding. Tumor growth was evaluated by measuring the tumor volumes as described above and determining the time tumors needed to grow from 50-60 mm^3^ to 100 mm^3^ (time to 100 mm^3^). The growth delay of irradiated tumors was calculated by subtracting the average time the nonirradiated tumors needed to reach 100 mm^3^ from the time that each irradiated tumor needed to reach 100 mm^3^.

## Statistical analysis

Statistical analysis and graph plotting were performed using GraphPad Prism 9 (La Jolla, CA, USA). Datasets were tested for normal distribution using the D’Agostino-Pearson normality test. Statistical significance was evaluated using ordinary one-way ANOVA with Tukey’s multiple comparisons test for normally distributed data and nonparametric one-way ANOVA (Kruskal‒Wallis test) for data without a normal distribution. A p value < 0.05 was considered statistically significant. Throughout the manuscript, the following symbols indicate statistical significance: * p < 0.05, ** p < 0.01, *** p < 0.001, and **** p < 0.0001. The sample size (n) for each experiment is presented in the figure legends and represents the number of biological replicates unless otherwise stated. All experiments were repeated at least two times.

# References

1. Schindelin J, Arganda-Carreras I, Frise E, Kaynig V, Longair M, Pietzsch T, et al. Fiji: an open-source platform for biological-image analysis. Nat Methods. 2012;9:676–82.

2. Jonkman JEN, Cathcart JA, Xu F, Bartolini ME, Amon JE, Stevens KM, et al. An introduction to the wound healing assay using live-cell microscopy. Cell Adh Migr. 2014;8:440–51.

3. Franken NAP, Rodermond HM, Stap J, Haveman J, van Bree C. Clonogenic assay of cells in vitro. Nat Protoc. 2006;1:2315–9.
